# Patients With Atypical Symptoms of Depression Respond Better to Lithium Augmentation Than Patients Without Atypical Symptoms

**DOI:** 10.1111/acps.70127

**Published:** 2026-07-17

**Authors:** Hannah Buchbauer, Mazda Adli, Peter Schlattmann, Maximilian Berger, Tom Bschor, Thomas Stamm, Joachim Behr, Roland Ricken, Pichit Buspavanich

**Affiliations:** ^1^ Department of Psychiatry and Neuroscience Campus Charité Mitte, Charité—Universitätsmedizin Berlin Berlin Germany; ^2^ Department of Psychiatry and Psychotherapy Fliedner Klinik Berlin Berlin Germany; ^3^ Department of Statistics, Informatics and Data Science Jena University Hospital Jena Germany; ^4^ Charité Center for Prevention, Health and Human Sciences, Research Unit Gender in Medicine Charité—Universitätsmedizin Berlin Berlin Germany; ^5^ Department of Psychiatry and Psychotherapy Technical University of Dresden Medical School Dresden Germany; ^6^ Faculty of Medicine and Psychology Brandenburg Medical School Theodor Fontane Neuruppin Germany; ^7^ Department of Psychiatry, Psychotherapy and Psychosomatics Brandenburg Medical School Theodor Fontane Neuruppin Germany; ^8^ Faculty of Health Sciences Brandenburg, Joint Faculty of the University of Potsdam, Brandenburg University of Technology Cottbus‐Senftenberg and Brandenburg Medical School Potsdam Germany; ^9^ Research Department of Experimental and Molecular Psychiatry, Department of Psychiatry and Psychotherapy Campus Charité Mitte, Charité—Universitätsmedizin Berlin Berlin Germany; ^10^ German Center for Mental Health Berlin Germany

**Keywords:** antidepressant therapy, atypical features, atypicality, major depressive disorder, response, treatment resistant depression

## Abstract

**Introduction:**

Understanding the differences in treatment response between subtypes of major depressive disorder (MDD) may help to prevent lengthy trial‐and‐error processes by identifying effective treatment options. The aim of the study was to investigate the impact of atypical features of MDD (MDD‐A) on the response to lithium augmentation (LA).

**Methods:**

In a multicenter prospective cohort study, response to LA was compared between patients with atypical symptoms of MDD (MDD‐A, *n* = 24) versus patients without atypical symptoms (MDD‐nA; *n* = 94) using a Cox regression analysis.

**Results:**

A total of 62.5% of patients with MDD‐A and 36.2% of patients with MDD‐nA responded to LA. Analysis adjusted for age, sex, sufficient lithium serum concentration, and symptom severity at baseline showed a significantly higher probability of patients with atypical symptoms to respond to LA (HR 2.20, *p* = 0.02).

**Conclusion:**

This is the first study investigating lithium‐response in patients with MDD‐A. The MDD‐A subtype may help to predict response to LA.

**Trial Registration:** EudraCT number: 2008‐004182‐26; ClinicalTrials.gov identifier: DRKS00026205

## Key Points

- Significant outcomes
  ○ Patients with major depressive disorder (MDD) with atypical symptoms respond significantly better to treatment with LA than patients with MDD without atypical symptoms.
- Limitations
  ○ Atypical symptoms were diagnosed retrospectively based on self‐reported measures.
  ○ There was no control group.

## 1 Introduction

Major depressive disorder (MDD) has an international lifetime prevalence of 16%–20% [1, 2]. More than 30% of affected patients do not achieve remission after receiving adequate pharmacotherapeutic treatment [3, 4]. In consequence, many patients must undergo a lengthy trial‐and‐error process [5], which can lead to a high illness burden and chronicity of the disorder. For patients who do not respond sufficiently to treatment with first‐line antidepressants, lithium can additionally be given to augment the antidepressant effect. With a mean response rate of 45% [6], lithium augmentation (LA) is among the second step strategies with the best evidence for effectiveness and is recommended by national and international treatment guidelines [7, 8, 9].

Research on antidepressant pharmacotherapy may study relevant biomarkers, as well as the symptom profile of an individual patient for determining the optimal treatment [10]. Since there are various types of antidepressants with differing biological mechanisms of action, certain antidepressants may be more advantageous for a particular subgroup of patients. For instance, the sedative effects of mianserin are of special benefit for patients with insomnia [11, 12], while light therapy is indicated for patients with seasonal affective disorder [13].

Based on the symptom profile, the DSM‐V differentiates specifiers of MDD, such as melancholic depression or atypical depression (MDD‐A) [14]. This subtype was introduced into the DSM due to findings of superior efficacy of monoamine oxidase inhibitors (MAOI) compared with tricyclic antidepressants (TCAs) in this subset of patients [15], underlining the relevance of subtypes for the selection of adequate treatment.

MDD‐A is defined by patients experiencing mood reactivity and at least two of the following symptoms: increased appetite or weight gain, hypersomnia, leaden paralysis, and interpersonal rejection sensitivity [14]. The prevalence of MDD‐A varies by sample and definition, ranging from 7% to 66% with a particular prevalence seen in inpatients [16, 17, 18, 19, 20, 21, 22, 23]. MDD‐A has previously been found to be associated with a higher prevalence in women compared with men [24] and a family history of unipolar and bipolar depression [25, 26]. Though a superior response to MAOI compared with TCAs is described for MDD‐A [27], there are thus far no studies investigating the response of patients with MDD‐A to LA. A connection between atypicality and bipolar disorder (BD) has previously been postulated [28, 29, 30, 31]. Considering this notion and the well‐established efficacy of lithium for BD [32], it is of interest to investigate the efficacy of LA for patients with MDD‐A.

## 2 Aim of the Study

To help bridge the data gap regarding the optimal treatment of patients with atypical depression and the potential benefit of LA, the current study investigates whether there is a difference in the response to LA between patients with atypical depression and those with depression without atypical symptoms.

## 3 Materials and Methods

The sample consisted of *N* = 118 patients from a multicentric naturalistic cohort study (registration in the German Clinical Trials Register, number DRKS00026205), of which *n* = 24 had MDD‐A and *n* = 94 had depression without atypical symptoms (MDD‐nA; see Figure 1 for a flow diagram of inclusion). Patients were recruited from 13 psychiatric departments of the Berlin Research Network on Depression between 2008 and 2020. The inclusion criteria were: minimum age of 18 years, diagnosis of MDD as confirmed by experienced clinicians and the Mini‐International Neuropsychiatric Interview (MINI), and indication for antidepressant pharmacotherapy. Patients were included if they showed an insufficient response to adequate treatment with one or more antidepressants. The indication for LA was assessed by the treating clinician. During the study, the antidepressant treatment was continued, with lithium added as an augmentative treatment. Patients were not included if they had a contraindication against LA, a diagnosis of dementia, an organic brain disorder, current substance abuse, or antisocial personality disorder.

**FIGURE 1:**
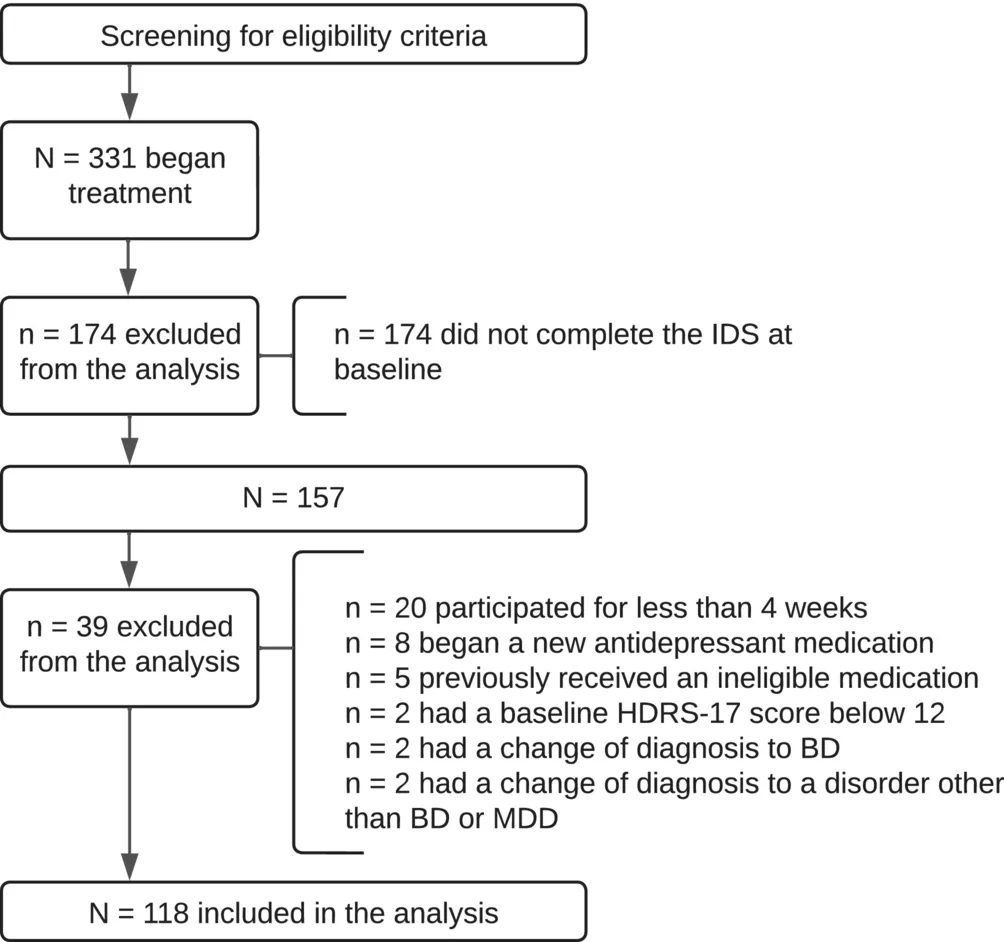
Flow diagram of inclusion. BD: bipolar disorder; HDRS‐17: Hamilton Depression Rating Scale 17; IDS: Inventory of Depressive Symptomatology; MDD: major depressive disorder.

In weekly visits, lithium serum level was measured, and further clinical data was collected. The severity of the current episode was measured with the Hamilton Depression Rating Scale (HDRS‐17) [33]. The 30‐item Inventory of Depressive Symptomatology Self Rating (IDS‐SR) [34] was used to measure atypical symptoms at baseline. In line with the DSM‐V criteria and previous publications on MDD‐A [35, 36], patients were diagnosed with MDD‐A if they experienced “mood reactivity” (Item 8) and two or more of the following additional symptoms: hypersomnia (Item 4), increased appetite or weight gain (Items 12 or 14), leaden paralysis (Items 20 or 30), rejection sensitivity (Items 6 or 29). A score of > 1 on each IDS‐SR‐item was considered as presence of the corresponding symptom, apart from item 8, where a score of < 1 corresponded to mood reactivity. Response was defined as a reduction of 50% or more in the HDRS‐17 sum score or a score below 9.

To test differences in time to response in days following the beginning of treatment with LA, a Cox regression analysis was performed using SPSS Version 29.0. The variables sex, age, HDRS‐17 sum score at baseline, and a sufficient lithium serum level were included in the regression model. Additionally, univariate Cox regression analyses were done to screen all clinical variables. Any variable with a significance of *p* < 0.05 was included in the regression model to control for confounding. Furthermore, group differences between MDD‐A and MDD‐nA regarding demographic and clinical variables were tested. Here, two‐sample *t*‐tests, Mann Whitney *U* tests, Welch two‐sample *t‐*tests, and two‐tailed chi‐squared tests were used as appropriate. For all analyses, the significance level was set at *p* < 0.05, and *p* < 0.1 was considered a trend.

## 4 Results

There were significant differences between MDD‐A and MDD‐nA in the severity of depression at baseline and endpoint (see Table 1), as well as in response. No significant group differences in demographic variables or remaining clinical variables were found. A trend was found for the number of hospitalizations (see Table 1). The Cox regression analysis of the time to response revealed that atypical symptoms significantly influenced the response to LA when controlling for the effects of age, sex, sufficient lithium serum concentration, and symptom severity at baseline. None of the other variables in the model had a significant effect on the response (see Table 2 and Figure 2).

**TABLE 1:** Comparison of demographic and clinical characteristics: Patients with MDD‐A vs. patients with MDD‐nA; *n* (%), mean [standard deviation].

	MDD‐A	MDD‐nA	*p*
Response	*n =* 15 (62.5)	*n =* 34 (36.2)	**0.019**
Age	*n =* 24, 47 [13.1]	*n =* 94, 46.8 [12.8]	0.891
Sex			0.867
Female	*n =* 15 (62.5)	*n =* 57 (60.6)	
Male	*n =* 9 (37.5)	*n =* 37 (39.4)	
Type of MDD			0.824
Single episode	*n =* 6 (25.0)	*n =* 22 (23.4)	
Recurrent episodes	*n =* 16 (66.7)	*n =* 52 (55.3)	
Duration of current episode			0.602
< 6 months	*n* = 12 (50.0)	*n* = 44 (46.8)	
> 6 months	*n* = 10 (41.7)	*n* = 47 (50.0)	
Mean duration of episodes			0.602
< 6 months	*n* = 10 (41.7)	*n* = 41 (43.6)	
> 6 months	*n* = 14 (58.3)	*n* = 45 (47.9)	
Psychotherapy In current episode			0.511
Yes	*n* = 18 (75.0)	*n* = 64 (68.1)	
No	*n* = 6 (25.0)	*n* = 30 (31.9)	
HDRS‐17 baseline score	*n =* 24, 18.9 [4.2]	*n =* 94, 22.1 [5.1]	**0.007**
HDRS‐17 score at study endpoint	*n =* 24, 8.4 [4.1]	*n =* 94,13.5 [6.8]	**< 0.001**
BMI at baseline	*n =* 21, 25.2 [4.0]	*n =* 90, 26.5 [5.6]	0.472
Charlson comorbidity score	*n =* 24, 0.2 [0.7]	*n =* 90, 0.2 [0.7]	0.906
Lithium level at study endpoint	*n =* 24, 0.7 [0.2]	*n =* 94, 0.7 [0.2]	0.683
Number of prior episodes	*n =* 22, 3.6 [4.4]	*n =* 74, 4.7 [6.2]	0.397
Number of hospitalizations	*n =* 24, 1.6 [1.9]	*n =* 90, 2.4 [2.9]	0.056
Number of affected relatives	*n =* 23, 1.4 [1.2]	*n =* 88, 1.3 [1.5]	0.697
Psychotropic comedication^a^			
SSRI	*n* = 14	*n* = 36	0.076
SNRI	*n* = 8	*n* = 36	0.654
TCA	*n* = 2	*n* = 16	0.291
NDRI	*n* = 0	*n* = 4	0.304
Valdoxan	*n* = 0	*n* = 6	0.204
Mirtazapine	*n* = 3	*n* = 19	0.387
MAOI	*n* = 0	*n* = 5	0.248
Atypical Antipsychotics	*n* = 5	*n* = 18	0.853
Antiepileptic drugs	*n* = 1	*n* = 10	0.330
Benzodiazepines	*n* = 7	*n* = 24	0.718
Low‐potency antipsychotics	*n* = 1	*n* = 9	0.396

*Note: N* = 118. In the case of a nonsignificant Levene test, the Welch two‐sample *t‐*test was used; in the case of nonnormal distribution, the Mann–Whitney *U* test was used; for categorical variables, the chi‐squared test was used. All significant predictors are in bold.

Abbreviations: BMI: body mass index; HDRS‐17: Hamilton Depression Rating Scale 17; MAOI: monoamine oxidase inhibitor; MDD‐A: major depressive disorder with atypical symptoms; MDD‐nA: major depressive disorder without atypical symptoms; NDRI: norepinephrine‐dopamine reuptake inhibitor; SD: standard deviation; SNRI: serotonin‐norepinephrine reuptake inhibitor; SSRI: selective serotonin reuptake inhibitor; TCA: tricyclic antidepressant.

^a^ Patients may have received more than one comedicationi.

**TABLE 2:** Cox regression analysis of time to response.

	Standard error	Hazard ratio	*p*	95% Ci	
				Lower	Upper
Atypical Symptoms	0.34	2.20	**0.024**	1.11	4.30
Age	0.01	1.01	0.453	0.99	1.03
Sex	0.33	1.55	0.182	0.81	2.95
Sufficient lithium concentration	0.74	4.24	0.051	1.00	18.05
HDRS‐17	0.03	0.99	0.755	0.93	1.05

*Note: N* = 118; *n* = 69 (58.5%) censored as nonresponders; Response: ≥ 50% reduction in HDRS‐17 score or HDRS score below 9. All significant predictors are in bold.

Abbreviation: HDRS‐17: Hamilton Depression Rating Scale 17.

**FIGURE 2:**
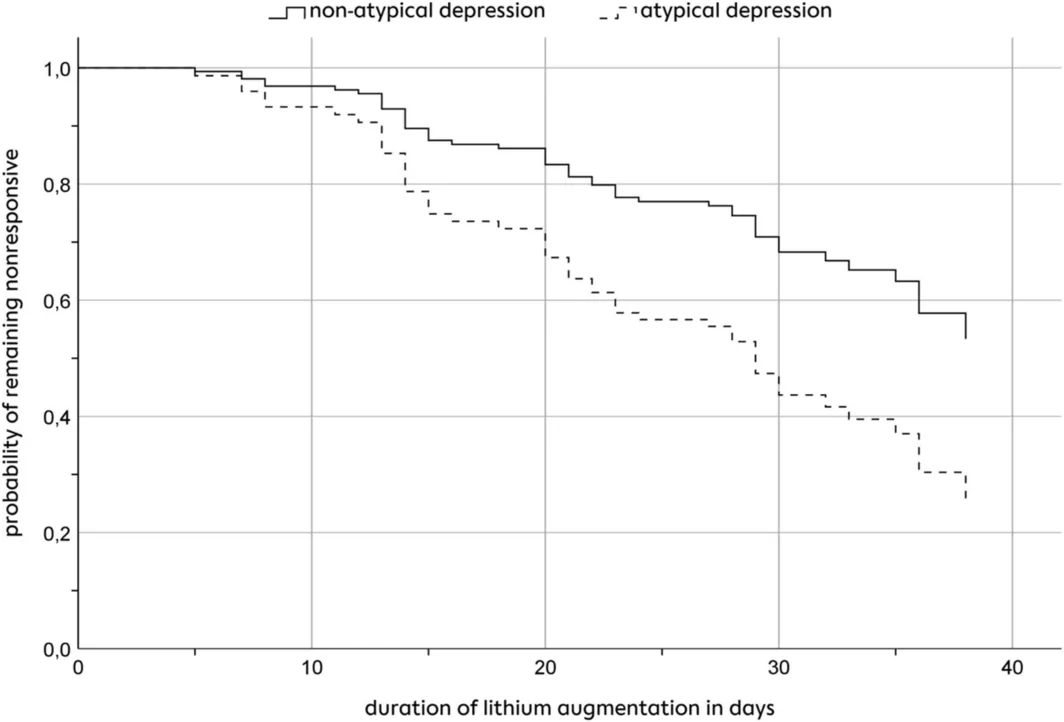
Cox regression curve. *N* = 118; Cox regression analysis of time to response between patients with nonatypical depression (solid line) and patients with atypical depression (dashed line).

## 5 Discussion

This is the first study investigating the efficacy of LA specifically in patients with atypical symptoms compared with patients without atypical symptoms. Patients with MDD‐A had a significantly higher probability of achieving response to LA than patients with MDD‐nA within a fixed time. This effect remained significant when controlling for sex, age, symptom severity at baseline, and sufficient lithium serum level.

Previous studies found differences in response to specific antidepressant monotherapy strategies in the MDD‐A subtype. Most evidence has been found for MAOIs as an effective treatment option for MDD‐A. This has been confirmed by a recent systematic review and network meta‐analysis by Fornaro et al. [27] including data of 21 randomized controlled trials (RCTs) investigating the pharmacological efficacy and tolerability of MDD‐A (*N* = 23 and *N* = 401). Regarding efficacy, 13 studies were included in the network meta‐analysis. The only treatment that was significantly more effective than placebo, as measured by the change in symptom severity, was phenelzine, a nonselective and irreversible MAOI. Among various antidepressants, phenelzine had the highest response probability and was also superior to the TCA imipramine. Therefore, the authors conclude that based on the current literature, phenelzine is the best option for MDD‐A, though it should be taken into consideration that MAOIs are not available for prescription in many countries, have many drug interactions, and require dietary restrictions. Noteworthy, data concerning LA were not included in the analyses.

In a systematic review and meta‐analysis, Ricken et al. [37] postulated a possible superiority of the nonselective and irreversible MAO‐I tranylcypromine in the treatment of MDD‐A based on three studies. These showed tranylcypromine to be significantly more effective than placebo [38] and imipramine [39, 40] in patients with atypical depression. However, two included studies examined only patients with BD and atypical symptoms. In both these studies, the sample included *n* = 28 patients with atypical symptoms, thus a sample size comparable to the current study. The third included study was conducted with a small sample of *N* = 16 patients. Another meta‐analysis investigating the effectiveness of the selective and irreversible inhibitor of MAO‐B selegiline in three studies (*n* = 136) found selegiline to be more effective than placebo in the treatment of MDD‐A [41]. Interestingly, one study found cognitive therapy to be similarly effective to MAOIs in the treatment of MDD‐A [42]. Husain et al. [43] studied electroconvulsive therapy in a randomized controlled trial and found patients with MDD‐A to be 2.6 times more likely to remit following treatment than patients with MDD‐nA, when adjusting for several clinical covariates. A recent observational study by Patarroyo‐Rodriguez et al. [44] with a sample of *N* = 52 patients found that patients with hypersomnia at baseline had a higher response rate to treatment with intravenous ketamine or intranasal esketamine. Of the eight patients that had both hypersomnia and hyperphagia, the majority responded to treatment. This study shows preliminary evidence for esketamine as a relevant treatment option for MDD‐A. However, the sample size is small, and the DSM‐V criteria were not used to diagnose MDD‐A. In summary, these studies confirm the relevance of MDD‐A as a phenotype that may have the potential to predict the response to specific antidepressant treatment strategies, as does the current study on LA.

Conversely, a study by Uher et al. [23] investigated the response of several subtypes of MDD (melancholic, atypical, anxious, and anxious‐somatizing) to treatment with the selective serotonin reuptake inhibitor (SSRI) escitalopram and the noradrenergic TCA nortriptyline in a larger sample. This study did not find any of the subtypes to predict the response to either of the antidepressants. Thus, current evidence suggests that these subtypes do not predict treatment response to SSRIs and noradrenergic TCAs. The underlying biological mechanisms of subtypes of MDD are not yet fully understood [45]. Hence, specific antidepressants may be more suitable to certain subtypes due to their mechanism of action, while other antidepressants might be similarly effective across different subtypes [46, 47]. Currently, literature indicates a preference for MAOIs, esketamine, and electroconvulsive therapy for the treatment of MDD‐A.

Several mechanisms of action of lithium are known that may in part explain its efficacy as an augmentative treatment of MDD‐A. Among them are immuno‐regulatory effects [48]. Immunotherapy is being studied in the treatment of MDD [49]. However, anti‐inflammatory treatment seems to have an effect on MDD only in patients with high levels of inflammation at baseline [50, 51]. Previous studies have found atypical depressive symptoms to be associated with higher inflammation [52, 53]. For instance, Zwiep et al. [54] suggest that patients with MDD‐A, who exhibited elevated C‐reactive protein levels, represent a distinct subgroup characterized by significant immuno‐metabolic dysregulation. Previous studies on lithium have found both pro‐inflammatory as well as anti‐inflammatory effects, depending on the experimental conditions [55, 56, 57]. The association of atypical symptoms with inflammation and anti‐inflammatory effects of lithium might have the potential to partially explain the current findings. Thus, clinical studies should be conducted to investigate whether the response to LA of patients with MDD‐A is modulated by changes in inflammatory markers.

There is an overlap in symptoms between patients with MDD‐A and those with BD, such as irritability and phases of brightened mood in patients with BD, similar to interpersonal rejection sensitivity and mood reactivity in patients with MDD‐A. It has been postulated that symptoms of MDD‐A could be signs of undiagnosed BD [29, 30]. This notion is supported by findings of a higher prevalence of atypicality in BD than in MDD [58], as well as a higher conversion of the diagnosis of MDD to BD in patients with MDD‐A than in patients with MDD‐nA [26]. A recent review confirmed that lithium remains the most effective prophylactic treatment for BD [32]. Studies have also shown lithium to be more effective for BD than for unipolar depression [59]. The group difference in response to LA between MDD‐A and MDD‐nA is in line with the notion of a connection between atypicality and BD [30, 31]. Common characteristics between patients with BD and patients with MDD‐A may partly explain better response to LA in patients with MDD‐A compared with those with MDD‐nA. In line with this hypothesis, Kraft et al. [60] found that in patients with MDD, a higher polygenic risk score for BD is associated with the response and remission following LA. In future research, it would be of interest to investigate whether a higher polygenic risk score for BD is associated with atypical symptoms.

Due to the relatively short observation period of 4 weeks, undiagnosed bipolarity cannot be entirely ruled out. Patients could experience manic or hypomanic phases in the future, thus switching to a diagnosis of BD and a future group difference in this aspect is possible. Nonetheless, aside from the diagnosis of unipolar MDD given by senior psychiatrists in psychiatric departments, the MINI was used to confirm the diagnosis of unipolar MDD and screen for previous or current (hypo‐) manic or mixed episodes.

Though atypical features were not diagnosed using a standardized diagnostic interview, most previous studies on atypical symptoms have used the IDS‐SR or a combination of the IDS‐SR with other rating scales to diagnose the atypical specifier of the DSM‐V [35, 36]. Consequently, a comparison can be drawn between the results of the present study and most previous studies on other antidepressant agents. However, when interpreting these findings, it should be noted that, in our study, patients with MDD‐A had less severe depression at baseline. The difference in response reflects an effect independent of severity. Nevertheless, residual confounding cannot be ruled out entirely. A key strength of this study is that experienced clinicians assessed and treated patients, ensuring comprehensive care for MDD. Further, using standardized symptom classification and including relevant covariates such as age, sex, baseline symptom severity, and sufficient lithium serum levels enhances the internal validity of the findings. However, it is important to acknowledge the study's limitations. First, the cross‐sectional design of the study may not be able to capture changes in specific symptoms over time. Second, the lack of a control group and randomization precludes causal interpretations. Third, the relatively small sample size—particularly within the MDD‐A subgroup—limits the generalizability of the findings.

## 6 Conclusion

This study suggests that LA can be a more effective treatment for patients with MDD who exhibit atypical symptoms. To the authors' knowledge, it is the first study investigating LA in patients with MDD‐A. The findings underscore the importance of symptom‐based phenotyping and highlight the clinical relevance of considering individual symptom profiles when selecting augmentation strategies. The guidelines for the treatment of MDD by the American Psychiatric Association [7] and the Institute for Clinical Systems Improvement [61] recommend treating patients with atypical features with MAOIs. Pending replication of the current results, LA may also be considered as a viable option, as well as esketamine and electroconvulsive therapy. Given the observational nature of the study and its limitations, further research is needed. Future investigations require replication of these findings in larger randomized controlled trials, as well as exploration of the biological underpinnings of the atypical symptom profile. Understanding how these mechanisms interact with lithium could help identify predictors of treatment response and advance precision medicine, leading to tailored interventions for MDD patients.

## Author Contributions

H.B. undertook the statistical analysis, conducted the literature searches, and wrote the first and final drafts of the manuscript. M.A. designed the study, wrote the protocol, recruited study subjects, supervised the project, provided critical feedback, and revised the final draft. P.S. supervised the statistical analysis. T.B., T.S., and J.B. recruited study subjects, undertook parts of the statistical analysis, provided literature searches, and critical feedback. R.R. designed the study, wrote the protocol, recruited study subjects, supervised the project, provided literature searches, and critical feedback. P.B. recruited study subjects, revised the manuscript until the final draft, supervised the project, undertook the statistical analysis, and managed the literature searches. All the authors contributed to and approved the final manuscript.

## Funding

This work was supported with an industry research grant by Lundbeck GmbH and Mood Disorders Research Group.

## Ethics Statement

The ALIA and ELIA studies were approved by the ethics committee of the Charité—University Medicine.

## Consent

All participants provided written informed consent.

## Conflicts of Interest

R.R. received a research grant and speaker honoraria from Aristo Pharma GmbH. H.B. receives the Elsa‐Neumann‐Scholarship of the state of Berlin for her doctoral studies. M.A. has received speaker honoraria from Johnson & Johnson, Gilead Sciences, Aristo, Pfizer, Biogen, MCI, Galapagos Biopharma. M.A. has been a consultant to or was active at an advisory board for Johnson & Johnson, Boehringer Ingelheim, Neuraxpharm, PATH, Hogan Lovells. M.A. has received grants or research support from Berlin University Alliance, Analytical Psychiatry Foundation, Lundbeck GmbH and the European Union's Horizon program. P.B. received research grants from the Berlin University Alliance, Federal Ministry of Education and Research, the Federal Ministry of Economic Affairs and Climate Action, and the European Union's Horizon program. The other authors declare no conflicts of interest.

## Data Availability

The data that support the findings of this study are available on request from the corresponding author. The data are not publicly available due to privacy or ethical restrictions.
